# Association of HER-2/CEP17 Ratio and HER-2 Copy Number With pCR Rate in HER-2-Positive Breast Cancer After Dual-Target Neoadjuvant Therapy With Trastuzumab and Pertuzumab

**DOI:** 10.3389/fonc.2022.819818

**Published:** 2022-03-04

**Authors:** Fanfan Li, Qian Ju, Cong Gao, Jingjing Li, Xiaolei Wang, Min Yan, Liying Zhang, Meiling Huang, Qihe Long, Xiangting Jin, Nanlin Li

**Affiliations:** ^1^ Department of Oncology, The Second Affiliated Hospital of Anhui Medical University, Hefei, China; ^2^ Department of Pathology, Xijing Hospital of Air Force Military Medical University, Xi ‘an, China; ^3^ Department of Breast Surgery, Xijing Hospital of Air Force Military Medical University, Xi ‘an, China

**Keywords:** breast cancer, HER-2 positive, IHC, FISH, dual-target neoadjuvant therapy

## Abstract

**Objective:**

To explore the correlation between HER-2 status and pathological complete response (pCR) in HER-2-positive breast cancer after dual anti-HER-2 neoadjuvant therapy with trastuzumab and pertuzumab.

**Methods:**

A total of 57 HER-2-positive breast cancer patients admitted to the Second Affiliated Hospital of Anhui Medical University and Xijing Hospital Affiliated to Air Force Military Medical University, between January 1, 2019 and September 30, 2020, were enrolled in this multicenter retrospective study. HER-2 status, including HER-2/CEP17 ratio and HER-2/cell number ratio, was detected by FISH. The correlation between HER-2 status/clinicopathological data and pCR was analyzed. The ROC curve was drawn to determine the cutoff value.

**Results:**

IHC assessment revealed 40 (70.18%) patients with IHC 3+ and 17 (29.82%) with IHC 2+. 41/57 (71.93%) patients achieved pCR. FISH revealed that the ratio of HER-2/chromosome 17 centromere (HER-2/CEP17) (p<0.001) and the ratio of HER-2/cell number (p<0.001) was significantly correlated with the pCR rate. ROC analysis showed that patients with an HER-2/CEP17 ratio ≥4.495 or HER-2/cell number ≥11.650 have a high pCR rate after dual anti-HER-2 neoadjuvant therapy, suggesting its predictive significance.

**Conclusion:**

The response to dual-targeted neoadjuvant therapy with trastuzumab and pertuzumab was adequate in hormone receptor-negative, HER-2-positive breast cancer patients. HER-2/CEP17 ratio and HER-2/cell number ratio were crucial for predicting efficacy.

## Introduction

Breast cancer has replaced lung cancer as the world’s most common malignant tumor that severely endangers women’s health (1). Human epidermal growth factor receptor-2 (HER-2)-positive breast cancer accounts for 20–25% of all breast carcinomas and has a poor prognosis and high recurrence rate (2). However, the development of the HER-2-targeting antibody has profoundly improved the outcome in HER-2-positive breast cancers (3).

As monoclonal anti-HER-2 drugs, trastuzumab and pertuzumab, act on two different domains of HER-2, respectively, and synergistically inhibit HER-2-positive breast cancer (4–8). Recently, several randomized trials have also investigated its efficacy in the neoadjuvant setting, with pathological complete response (pCR) as the primary endpoint. In the NeoSphere study, the pCR rate of trastuzumab combined with pertuzumab (dual anti-HER-2 therapy) combined with docetaxel was significantly higher than that in the single-target group (9). The five-year follow-up to the NeoSphere study confirmed that patients achieving pCR had longer disease-free survival (DFS) than those who did not achieve pCR. Despite the improvement of 45.8%, more than 50% of patients did not achieve pCR. Thus, screening patients with high drug sensitivity before neoadjuvant therapy is crucial in HER-2-positive breast cancer patients.

HER-2 is a transmembrane glycoprotein with tyrosine kinase activity that belongs to the epidermal growth factor receptor family. The amplification of the *HER-2* gene is the primary mechanism of HER-2 overexpression. The HER-2 status is necessary for anti-HER-2 treatment, based on immunohistochemistry (IHC) to assess the protein overexpression and *in-situ* hybridization (ISH) to assess gene amplification (10). However, both tests determined whether a tumor is a potential target for anti-HER-2 treatment response in the metastatic setting, which is only achieved in about 50% of cases, while >25% of women treated with neoadjuvant anti HER-2 therapy relapse within the first ten years of diagnosis (11). The predictive efficacy of HER-2 amplification and expression has not been studied in-depth. Currently, only foreign research data are available on the curative effect of neoadjuvant trastuzumab-based chemotherapy for HER-2 positive breast cancer. This retrospective analysis aimed to evaluate the correlation between HER-2 data and pCR of HER-2-positive breast cancer patients treated with dual-target neoadjuvant therapy of trastuzumab and pertuzumab.

## Material and Methods

### Patients

Patients with core biopsy confirmed HER-2-positive breast cancer, between January 1, 2019 and September 30, 2020, from the Second Affiliated Hospital of Anhui Medical University and Xijing Hospital Affiliated to Air Force Military Medical University, were enrolled in this retrospective study. All patients were treated with dual-target neoadjuvant therapy after primary tumor and axillary lymph node core biopsy confirmed. The inclusion criteria were as follows: (1) age 18–75-years-old, (2) HER-2-positive non-special type invasive breast cancer; (3) received dual anti-HER-2 neoadjuvant therapy with trastuzumab and pertuzumab; (4) written informed consent. The exclusion criteria were as follows: (1) male gender; (2) metastatic disease at diagnosis; (3) cases with incomplete clinical or pathological data and patients without surgical treatment.

The study protocol was approved by an independent ethics committee of the Second Affiliated Hospital of Anhui Medical University, and all participants provided informed consent (approval number: XY2021-023). The study was conducted in accordance with the Good Clinical Practice guidelines and the Declaration of Helsinki. All patients provided written informed consent before enrollment.

### Neoadjuvant Therapy Regimen

All patients were treated with three types of neoadjuvant chemotherapy combined with trastuzumab and pertuzumab regimen for 6–8 cycles after the diagnosis was confirmed by a puncture. Some patients were treated with single-agent docetaxel or paclitaxel combined with trastuzumab plus pertuzumab regimen (THP) for six cycles, while some were treated with docetaxel or paclitaxel combined with carboplatin plus trastuzumab plus pertuzumab regimen (TCbHP) treatment for six cycles. The other patients were treated with docetaxel or paclitaxel combined with trastuzumab plus pertuzumab regimen for four cycles before or after treatment with doxorubicin or epirubicin combined with cyclophosphamide regimen for four cycles (THP-A(E)C/A(E)C-THP). Surgery was performed after the completion of neoadjuvant treatment.

### ER and PR Detection

Estrogen receptor (ER) and progesterone receptor (PR) were detected by immunohistochemistry (IHC). These data were obtained from pathological reports of needle core biopsies and postoperative specimens and evaluated according to the 2018 ASCO/CAP guidelines using FDA-approved analysis (12). The ER and PR positivity is defined as nuclear staining of at least 1% of tumor cells (13).

### HER-2 Detection and Analysis

IHC and fluorescence *in situ* hybridization (FISH) were also used for HER-2 assessment. HER-2 positivity was defined as HER-2 IHC3+, or HER-2 IHC2+ but HER-2 FISH amplified. HER-2 IHC results come from the pathology report. HER-2 IHC score is positive (3+), suspicious (2+), or negative (0 or 1+) (12). IHC 2+ (equivocal) is invasive breast cancer with “weak to moderate complete membrane staining observed in >10% of tumor cells”. Next, we evaluated the expression of HER-2 in all invasive breast cancers through IHC. Typically, HER-2 IHC 3+ cases do not require further testing. All suspected HER-2 (IHC 2+) cases were evaluated by HER-2 FISH. In order to explore the predictive significance of the efficacy, we performed FISH on needle core biopsies before neoadjuvant treatment of all breast cancer patients. It was also performed on paraffinized tissue sections using the HER-2 DNA Dual Probe Kit (Linked-Biotech Pathology, Guangzhou, People’s Republic of China).Furthermore, the HER-2/CEP17 ratio ≥2.0 or an average HER-2 copy number ≥6.0/cell (the ratio of HER-2/cell number) was evaluated in FISH (12). HER-2 and CEP17signals of ≥20 nuclei of tumor cells within invasive tumor areas were measured to determine the HER-2/CEP17 ratio. If the HER-2/CEP17 ratio exceeded 2 or the absolute HER-2 copy number exceeded 6, tumors were considered to be HER-2 FISH amplified, according to the current ASCO/CAP guidelines.

### pCR

pCR was defined as no residual invasive cancer in the breast and axillary lymph nodes, irrespective of ductal carcinoma *in situ* residual components (ypT0/is ypN0).

### Statistical Analysis

Quantitative data were assessed for normality using the Shapiro–Wilk (S-W) method; t-test was used for comparison with normal distribution; the rank-sum test was used for comparison with non-normal distribution. Categorical variables are compared by Fisher’s exact test or χ^2^ test. The multivariate analysis was carried out using multivariable Logistic Regression model. And multivariable Logistic Regression model was fit by a backward selection method with an alpha =0.20 removal criteria. The receiver operator characteristic (ROC) curve was used to determine the cutoff value of the HER-2/CEP17 ratio and HER-2/cell number ratio in FISH detection for the prognostic value. The value corresponding to the maximum Youden index is chosen as the optimal cutoff value. All statistical analyses were conducted using SPSS 23.0 statistical software to analyze the data. The p-values were two-sided, and p<0.05 was considered statistically significant.

## Results

### Baseline Characteristics

A total of 57 patients, aged 48.46-years-old, were included in this study. The baseline characteristics were listed in **Table 1**. 29/57 (50.88%) patients were ER-positive, and 25/57 (43.86%) patients were PR-positive. Patients with pretreatment clinical T stages of T1, T2, and T3 accounted for 4/57 (7.02%), 39/57 (68.42%), and 14/57 (24.56%) patients, respectively. 36/57 (63.16%) patients had pretreated lymph nodes. Among them, 1 (1.75%), 47 (82.46%), and 9 (15.79%) patients had pretreatment puncture pathology grade 1, 2, and 3, respectively. In terms of chemotherapy regimen, 36/57 (63.16%) patients received anthracycline combined with taxane regimen [A(E)C-T/T-A(E)C], 11 (19.30%) patients received taxane combined with carboplatin regimen (TCb), and 10 (17.54%) patients received single taxane regimen (T) treatment.

**Table 1:** Baseline characteristics and pCR of 57 patients.

	Total (n=57)		pCR				pCR rate	t/χ^2^	p value
			pCR (n=41)		Non-pCR (n=16)				
Age	48.46 ± 10.89		48.37 ± 11.37		48.69 ± 9.89			0.099	0.921
Pretreatment clinical T staging									
cT1	4	0.07	4	0.10	0	0.00	1.00	28.472	<0.001
cT2	39	0.68	28	0.68	11	0.69	0.72		
cT3	14	0.25	9	0.22	5	0.31	0.64		
Pretreatment axillary lymph node status									
Positive	36	0.63	25	0.61	11	0.69	0.69	0.299	0.762
Negative	21	0.37	16	0.39	5	0.31	0.76		
Chemotherapy									
A(E)C-T/T-A(E)C	36	0.63	25	0.61	11	0.69	0.69	0.901	0.483
TCb	11	0.19	8	0.20	3	0.19	0.73		
T	10	0.18	8	0.20	2	0.13	0.80		
Pretreatment puncture pathology grading									
1	1	0.02	0	0.00	1	0.06	0.00	30.959	<0.001
2	47	0.82	35	0.85	12	0.75	0.74		
3	9	0.16	6	0.15	3	0.19	0.67		
ER									
Positive	29	0.51	15	0.37	14	0.88	0.52	11.937	0.001
Negative	28	0.49	26	0.63	2	0.13	0.93		
PR									
Positive	25	0.44	12	0.29	13	0.81	0.48	12.630	<0.001
Negative	32	0.56	29	0.71	3	0.19	0.91		
HER-2 IHC results									
2+	17	0.30	11	0.27	6	0.38	0.65	0.626	0.523
3+	40	0.70	30	0.73	10	0.63	0.75		

### HER-2 Status

IHC assessment revealed that 40 (70.18%) patients were IHC 3+ and 17 (29.82%) patients were IHC 2+. IHC 2+ was further confirmed with positive FISH results.

Among 57 patients, the average HER-2/CEP17 ratio was 5.55, the median was 5.25 (range:1.04–15.60), and the average of HER-2 copies/cell (HER-2 signal/cell number) was 15.08, and the median was 15.24 (range: 2.03–25.00). The average HER-2/CEP17 ratio among HER-2 IHC2+ patients and HER-2 IHC3+ patients was 4.17 and 6.23, respectively, and the difference was statistically significant (Z=-9.979, p<0.001). The average of HER-2 copies/cell among HER-2 IHC2+ patients and HER-2 IHC3+ patients was 11.80 and 16.68, respectively, and the difference was statistically significant (Z=-7.604, p<0.001).

### pCR and Its Clinicopathological Association

Among 57 patients, 41 (71.93%) achieved pCR. The pCR rate for ER-negative and ER-positive subgroups was 92.86% (26/28) and 51.72% (15/29), respectively, and the difference was statistically significant (χ^2^ = 11.937, p=0.001). On the other hand, the pCR rate of PR-negative and PR-positive patients was 90.63% (29/32) and 48.00% (12/25), significantly, and the difference was statistically significant (χ^2^ = 12.630, p<0.001). The pCR rates of pretreatment T clinical stages T1, T2, and T3 were 100.00% (4/4), 71.74% (28/39), and 64.29% (9/14), respectively, and the difference was statistically significant (χ^2^ = 28.472, p<0.001). The pCR rates of pretreatment puncture pathology grade 1, 2, and 3 were 0.00% (0/1), 74.47% (35/47), and 66.67% (6/9), respectively, and the difference was statistically significant (χ^2^ = 30.959, p<0.001). However, There was no statistical correlation between patient age, pretreatment lymph node status and neoadjuvant chemotherapy regimens and pCR rates (**Table 1**).

Multivariate analysis showed that ER and PR are significant predictors of pCR (**Table 2**). ER-negative tumors had significantly higher pCR rates compared to ER-positive tumors (odds ratio (OR)=0.074, 95% CI: 0.008–0.687, p=0.022). Compared to PR-positive patients, PR-negative patients had a significantly higher pCR rates (OR=0.179, 95% CI: 0.036–0.897, p=0.036). In multivariate analysis, pretreatment, clinical T stage, and pretreatment pathology were not associated with high pCR rates. It should be noted that 1)there was a strong correlation between HER-2 IHC results with HER2/CEP17 ratio and HER2 copy number;but in this study, there was no statistical correlation between HER-2 IHC results and pCR (p=0.523) and 2) it might be related to the small sample size of this study, and we will actively expand the sample size in future research. Therefore HER2/CEP17 ratio and HER2 copy number were not included in the multivariate model.

**Table 2:** Multivariate logistic analysis of pCR.

	Uni *p* value	Multi					
		*OR*	*β*	*SD*	*Wald χ^2^*	*95%CI*	*p* value
ER	0.001		-2.598	1.134	5.251	0.008-0.687	0.022
Negative		1					
Positive		0.074					
PR	<0.001		-1.721	0.823	4.375	0.036-0.897	0.036
Negative		1					
Positive		0.179					

pCR is assigned a value of 1, and non-pCR is assigned a value of 0; ER-negative is assigned a value of 0, and ER-positive is assigned a value of 1; PR-negative is assigned a value of 0, and PR-positive is assigned a value of 1; the puncture pathology grades 1, 2, and 3 are assigned as 1, 2, and 3, respectively).

### HER-2 Status and PCR

The pCR rate of patients with HER-2 protein overexpression (IHC 3+) was 75.00% (30/40). Conversely, the pCR rate of patients with HER-2 IHC 2+ and *HER-2* gene amplification detected by FISH was 64.71% (11/17) and no statistically significant difference was detected in the pCR between IHC 2+ (HER-2 FISH positive) and 3+ patients (χ^2^ = 0.626, p=0.523).

Increased HER-2/CEP17 ratio and HER-2 copy number/cell were associated with a high rate of pCR. Among 41 patients that reached pCR, the average HER-2/CEP17 ratio was 7.27, and the median was 5.85 (range: 3.59–15.60). The average HER-2/CEP17 ratio in 16 patients did not reach pCR was 3.98, the median was 4.17 (range: 1.04–7.35), and the difference was statistically significant (Z=-4.973, p<0.001). The average number of HER-2 copies/cell (HER-2 signal/cell number) in 41 patients with pCR was 17.13, and the median was 16.10 (range: 10.83–25.00); the average HER-2 copy number in 16 patients with no pCR was 10.55, and the median was 10.00 (range: 2.03–19.27), and the difference was statistically significant (Z=-10.838, p<0.001) (**Table 3**).

**Table 3:** FISH and pCR results.

	Non-pCR (N=16)	PCR (N=41)	*Z* value	*p* value
HER-2/CEP17 ratio			-4.973	<0.001
Range	1.04-7.35	3.59-15.60		
Average	3.98	7.27		
Median	4.17	5.85		
HER-2 signal/cell number			-10.838	<0.001
Range	2.03-19.27	10.83-25.00		
Average	10.55	17.13		
Median	10.00	16.10		

**Figure 1:**
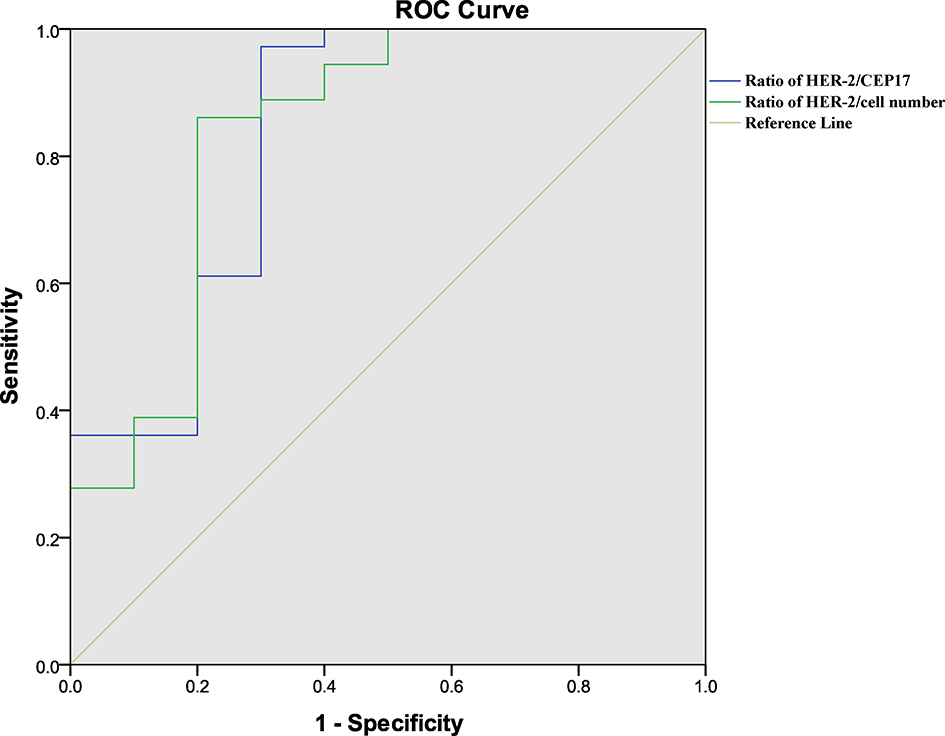
Results of ROC analysis. (green) ROC analysis of the ratio of HER-2/CEP17 with pCR. (blue) ROC analysis of the ratio of HER-2/cell number with pCR. p<0.05 was considered statistically significant.

The ROC curve (**Figure 1**) determined the cutoff value of HER-2/CEP17 ratio in FISH detection, which was 4.495 (area under the curve (AUC)=0.764, p=0.001), and the cutoff value of HER-2 copy number per cell was 11.650 (AUC=0.814, p<0.001).

According to the HER-2 copy number cutoff value, the patients were divided into high and low groups. Among ER-positive patients, the pCR rate of patients with high and low HER-2 copy number was 72.73% (16/22) and 14.28% (2/14) respectively, and the difference was statistically significant (χ^2^ = 11.688, p=0.002). Among ER-negative patients, 27 had high HER-2 copy number, and only 1 patient did not reach pCR; moreover, only 1 patient had low HER-2 copy number and did not reach pCR, and the difference was not statistically significant (p=0.071). Among the HER-2 IHC 2+ patients, 12 with high HER-2 copy number achieved pCR, while the pCR rate of patients with low HER-2 copy number was only 11.11% (1/9), and the difference was statistically significant (p<0.001). Among HER-2 IHC 3+ patients, the pCR rate of patients with high and low HER-2 expression was 81.08% (30/37) and 16.67% (1/6), respectively, and the difference was statistically significant (p=0.004).

According to the cutoff value of the HER-2/CEP17 ratio, patients were divided into high and low groups. Among ER-positive patients, the pCR rate of patients with high and low HER-2/CEP17 ratio was 83.33% (15/18) and 16.67% (3/18), respectively, and the difference was statistically significant (χ^2^ = 7.111, p=0.018). Among ER-negative patients, the pCR rate of patients with high and low HER-2/CEP17 ratio was 94.12% and 90.91% (10/11), respectively, and the difference was not statistically significant (p=1.000). Among the HER-2 IHC 2+ patients, 8 patients with a high HER-2/CEP17 ratio achieved pCR, and the pCR rate of patients with low HER-2/CEP17 ratio was 38.46% (5/13), with a statistically significant difference (p=0.007). In HER-2 IHC3+ patients, the pCR rate of patients with high and low HER-2/CEP17 ratio was 80.56% (29/36) and 28.57% (2/7), and the difference was statistically significant (χ^2^ = 7.872, p=0.012).

## Discussion

Breast cancer is a heterogeneous disease with diverse phenotypes. About 20–25% of invasive breast cancers have *HER-2* gene amplification or overexpression. Both early and metastatic breast cancers with HER-2-positive disease should be treated with a regimen containing anti-HER-2 therapy. The preoperative neoadjuvant therapy containing HER-2-targeted drugs for locally advanced HER-2-positive breast cancer patients reduces the size of primary tumors and axillary tumors, provides patients with surgical opportunities, improves patient prognosis, and identifies patients who may benefit from treatment or who do not respond satisfactorily (14, 15).

Presently, the data of dual-target neoadjuvant therapy in the Asian population is not sufficient. In various clinical studies (9, 16), the Asian population only accounts for about 20%, and the majority of subjects are Caucasians. Pertuzumab has been on the market in China for only 2 years, and no large-scale clinical data on dual-target neoadjuvant therapy in China are available. This is a small-sample real-world study of dual-target neoadjuvant therapy that provides data for domestic patients with HER-2-positive breast cancer adopting dual-target neoadjuvant therapy.

Dual-target neoadjuvant therapy with trastuzumab and pertuzumab blocked homodimer and heterodimer active model of HER-2, blocking the downstream signal transduction and showing promising efficacy against HER-2 positive breast cancer (17–21). In addition to the recurrence rate, the overall survival, DFS, and other indicators for evaluating the efficacy of adjuvant therapy, pCR is a major index for evaluating the efficacy of patients receiving neoadjuvant therapy. In the NeoSphere study, neoadjuvant therapy with trastuzumab, pertuzumab, and docetaxel increased the pCR rate from 24% to 46%, indicating long-term survival benefits (22). The current study represented 71.93% pCR rates for HER-2-positive breast cancers patients, which was obviously higher than the reported data. This phenomenon could be attributed to the following reasons. First, 82.46% of patients used the dual-drug chemotherapy (23.40% of patients used the TCb regimen chemotherapy, 76.60% of patients used the A(E)C-T/T-A(E)C regimen chemotherapy), which improved the efficacy. Second, the patients in this study received 6–8 cycles of neoadjuvant therapy, which was higher than the four cycles of neoadjuvant therapy in the NeoSphere study. Compared to the current pCR of about 56–60% in other studies (23–25), the pCR rate in the present study is higher, which might be related to changes in HER-2 pathological evaluation criteria. All patients in this study had onset after 2019, and the 2018 ASCO/CAP guidelines (12) were used to assess the HER-2 status, while most of the current studies are based on the 2015 ASCO/CAP guidelines to assess HER-2 status. The 2018 ASCO/CAP guidelines defined group2 as HER-2-positive in the original guidelines as negative. The new version of the guidelines may have excluded some patients with poor efficacy of anti-HER-2 therapy. In addition, since this was a small sample size retrospective study, there may be population selection bias. We will continue to follow up to observe the long-term survival data and confirm the long-term benefits of a high pCR rate.

Hormone receptor (HR) expression also has a predictive significance for the efficacy of neoadjuvant therapy. In this study, the pCR rate of HR-negative patients was higher than that in positive patients. Similarly, the NeoSphere study showed improved pCR in HR-negative patients (9). André et al. claimed that the crosstalk between HRs and HER-2 signaling pathways might be the putative reason for the low pCR of HR-negative patients (26). Some studies have shown that the pCR benefits of HR-negative patients are correlated with long-term survival benefits (27).

The evaluation of HER-2 status is significant for selecting anti-HER-2 therapy of patients. Reportedly, the consistency of IHC and FISH in assessing HER-2 status was >80%. The inconsistency between the two occurred when HER-2 IHC 2+ (28). Previous studies also showed that IHC 2+ (FISH-positive) patients have a lower pCR rate, which was consistent with our results (29, 30). In the current study, patients with HER-2 IHC 3+ had higher pCR rates than those with IHC ≤2+ (FISH-positive), but the difference was not statistically significant. This phenomenon could be attributed to the small sample size. An accurate prediction of anti-HER-2 efficacy would guide the clinical study (31, 32). According to previous data, the ratio of HER-2/CEP17 in FISH predicts the efficacy of trastuzumab-based anti-HER-2 neoadjuvant therapy and long-term prognosis. The pCR rate was high when the HER-2/CEP17 ratio ​​was higher than 6.0 and 7.0 (33, 34). In another study (23), the critical value that affects the pCR rate is set as HER-2/CEP17 = 4.5 and HER-2/cell number=14. In the current study, the ratio of HER-2/CEP17 and the ratio of HER-2/cell number had a specific correlation with the pCR rate. Patients with an HER-2/CEP17 ratio ≥4.49 or HER-2/cell number ≥11.650 have a high pCR rate after dual anti-HER-2 neoadjuvant therapy, suggesting its predictive significance. The cutoff value obtained from the analysis was further validated in ER-positive, HER-2 IHC 2+ (FISH-positive), and 3+ patients. However, the cutoff value was invalid, which might be related to the pCR rate of ER-negative patients (93%). Nonetheless, evidence from large sample size and its long-term prognostic benefits need to be further explored.

This study has some limitations. The sample size of this study was small, and the follow-up time was short. Since this was a retrospective study, the data were obtained from two hospitals, and pertuzumab was launched late in China (a realistic scenario is that pertuzumab was not available in mainland China: pertuzumab has not been approved for marketing until December 17, 2018, and available for purchase since March 19, 2019, and officially enter hospital system since January 1, 2020). Statistical methods were used to make up to increase the credibility of this study. The sample size would be expanded based on a multicenter study to verify the current findings in the future study. We will also continue to follow up all the patients to observe their long-term prognosis. This study aimed to provide new ideas for the efficacy prediction of neoadjuvant targeted therapy for HER-2-positive breast cancer in the dual-targeted therapy era.

In conclusion, the response to dual-targeted neoadjuvant therapy with trastuzumab and pertuzumab was better in hormone receptor-negative, HER-2 positive breast cancer patients. Patients with a HER-2/CEP17 ratio ≥4.49 or HER-2/cell number ≥11.650 have a high pCR rate, indicating its predictive value.

## Data Availability Statement

The original contributions presented in the study are included in the article/supplementary material. Further inquiries can be directed to the corresponding authors.

## Ethics Statement

The studies involving human participants were reviewed and approved by independent ethics committee of the Second Affiliated Hospital of Anhui Medical University. The patients/participants provided their written informed consent to participate in this study. Written informed consent was obtained from the individual(s) for the publication of any potentially identifiable images or data included in this article.

## Author Contributions

FL contributed to provided financial support. FL and NL contributed to conception, design, administrative support, revised the important knowledge content of the study and ensured the submission adheres to all journal requirements. QJ organized the database, performed the statistical analysis and wrote the first draft of the manuscript. FL, QJ and CG wrote sections of the manuscript. JL, XW, MY, LZ, MH, QL and XJ contributed data acquisition and analysis tools. All authors contributed to the article and approved the submitted version.

## Funding

This study was funded by the Natural Science Research Project of the Department of Education of Anhui Province (KJ2019A0258).

## Conflict of Interest

The authors declare that the research was conducted in the absence of any commercial or financial relationships that could be construed as a potential conflict of interest.

## Publisher’s Note

All claims expressed in this article are solely those of the authors and do not necessarily represent those of their affiliated organizations, or those of the publisher, the editors and the reviewers. Any product that may be evaluated in this article, or claim that may be made by its manufacturer, is not guaranteed or endorsed by the publisher.

## References

[1] Sung H Ferlay J Siegel RL Laversanne M Soerjomataram I Jemal A. Global Cancer Statistics 2020: Globocan Estimates of Incidence and Mortality Worldwide for 36 Cancers in 185 Countries. CA Cancer J Clin (2021) 71(3):209–49. doi: 10.3322/caac.21660(10.3322/caac.21660 33538338

[2] Slamon DJ Clark GM Wong SG Levin WJ Ullrich A McGuire WL. Human Breast Cancer: Correlation of Relapse and Survival With Amplification of the Her-2/Neu Oncogene. Science (1987) 235(4785):177–82. doi: 10.1126/science.3798106 3798106

[3] Witton CJ Reeves JR Going JJ Cooke TG Bartlett JM. Expression of the Her1-4 Family of Receptor Tyrosine Kinases in Breast Cancer. J Pathol (2003) 200(3):290–7. doi: 10.1002/path.1370 12845624

[4] Perez EA Romond EH Suman VJ Jeong JH Sledge G Geyer CE Jr. Trastuzumab Plus Adjuvant Chemotherapy for Human Epidermal Growth Factor Receptor 2–Positive Breast Cancer: Planned Joint Analysis of Overall Survival From Nsabp B-31 and Ncctg N9831. J Clin Oncol (2014) 32(33):3744–52. doi: 10.1200/jco.2014.55.5730 PMC4226805 25332249

[5] Marty M Cognetti F Maraninchi D Snyder R Mauriac L Tubiana-Hulin M. Randomized Phase Ii Trial of the Efficacy and Safety of Trastuzumab Combined With Docetaxel in Patients With Human Epidermal Growth Factor Receptor 2–Positive Metastatic Breast Cancer Administered as First-Line Treatment: The M77001 Study Group. J Clin Oncol (2005) 23(19):4265–74. doi: 10.1200/jco.2005.04.173 15911866

[6] O’Hagan D Schaffrath C Cobb SL Hamilton JT Murphy CD. Biochemistry: Biosynthesis of an Organofluorine Molecule. Nature (2002) 416(6878):279–80. doi: 10.1038/416279a 11907567

[7] Scheuer W Friess T Burtscher H Bossenmaier B Endl J Hasmann M. Strongly Enhanced Antitumor Activity of Trastuzumab and Pertuzumab Combination Treatment on Her2-Positive Human Xenograft Tumor Models. Cancer Res (2009) 69(24):9330–6. doi: 10.1158/0008-5472.CAN-08-4597 19934333

[8] Nahta R Hung M-C Esteva FJ. The Her-2-Targeting Antibodies Trastuzumab and Pertuzumab Synergistically Inhibit the Survival of Breast Cancer Cells. Cancer Res (2004) 64(7):2343–6. doi: 10.1158/0008-5472.Can-03-3856 15059883

[9] Gianni L Pienkowski T Im Y-H Roman L Tseng LM Liu MC. Efficacy and Safety of Neoadjuvant Pertuzumab and Trastuzumab in Women With Locally Advanced, Inflammatory, or Early Her2-Positive Breast Cancer (Neosphere): A Randomised Multicentre, Open-Label, Phase 2 Trial. Lancet Oncol (2012) 13(1):25–32. doi: 10.1016/s1470-2045(11)70336-9 22153890

[10] Recommended by Breast Cancer Expert P. Guideline for Her2 Detection in Breast Cancer, the 2019 Version. Zhonghua Bing Li Xue Za Zhi (2019) 48(3):169–75. doi: 10.3760/cma.j.issn.0529-5807.2019.03.001 30831640

[11] Perez EA Romond EH Suman VJ Jeong JH Sledge G Geyer CE Jr. Trastuzumab Plus Adjuvant Chemotherapy for Human Epidermal Growth Factor Receptor 2-Positive Breast Cancer: Planned Joint Analysis of Overall Survival From Nsabp B-31 and Ncctg N9831. J Clin Oncol: Off J Am Soc Clin Oncol (2014) 32(33):3744–52. doi: 10.1200/jco.2014.55.5730 PMC4226805 25332249

[12] Wolff AC Hammond MEH Allison KH Harvey BE Mangu PB Bartlett JMS. Human Epidermal Growth Factor Receptor 2 Testing in Breast Cancer: American Society of Clinical Oncology/College of American Pathologists Clinical Practice Guideline Focused Update. J Clin Oncol: Off J Am Soc Clin Oncol (2018) 36(20):2105–22. doi: 10.1200/jco.2018.77.8738 29846122

[13] Hammond ME Hayes DF Dowsett M Allred DC Hagerty KL Badve S. American Society of Clinical Oncology/College of American Pathologists Guideline Recommendations for Immunohistochemical Testing of Estrogen and Progesterone Receptors in Breast Cancer. J Clin Oncol: Off J Am Soc Clin Oncol (2010) 28(16):2784–95. doi: 10.1200/jco.2009.25.6529 PMC2881855 20404251

[14] von Minckwitz G Huang CS Mano MS Loibl S Mamounas EP Untch M. Trastuzumab Emtansine for Residual Invasive Her2-Positive Breast Cancer. N Engl J Med (2019) 380(7):617–28. doi: 10.1056/NEJMoa1814017 30516102

[15] Modi S Saura C Yamashita T Park YH Kim SB Tamura K. Trastuzumab Deruxtecan in Previously Treated Her2-Positive Breast Cancer. N Engl J Med (2020) 382(7):610–21. doi: 10.1056/NEJMoa1914510 PMC7458671 31825192

[16] Schneeweiss A Chia S Hickish T Harvey V Eniu A Waldron-Lynch M. Long-Term Efficacy Analysis of the Randomised, Phase Ii Tryphaena Cardiac Safety Study: Evaluating Pertuzumab and Trastuzumab Plus Standard Neoadjuvant Anthracycline-Containing and Anthracycline-Free Chemotherapy Regimens in Patients With Her2-Positive Early Breast Cancer. Eur J Cancer (2018) 89:27–35. doi: 10.1016/j.ejca.2017.10.021 29223479

[17] Ignatov T Gorbunow F Eggemann H Ortmann O Ignatov A. Loss of Her2 After Her2-Targeted Treatment. Breast Cancer Res Treat (2019) 175(2):401–8. doi: 10.1007/s10549-019-05173-4 30806922

[18] Murthy RK Raghavendra AS Hess KR Fujii T Lim B Barcenas CH. Neoadjuvant Pertuzumab-Containing Regimens Improve Pathologic Complete Response Rates in Stage Ii to Iii Her-2/Neu-Positive Breast Cancer: A Retrospective, Single Institution Experience. Clin Breast Cancer (2018) 18(6):e1283–8. doi: 10.1016/j.clbc.2018.07.008 30077429

[19] Swain SM Miles D Kim SB Im YH Im SA Semiglazov V. Pertuzumab, Trastuzumab, and Docetaxel for Her2-Positive Metastatic Breast Cancer (Cleopatra): End-Of-Study Results From a Double-Blind, Randomised, Placebo-Controlled, Phase 3 Study. Lancet Oncol (2020) 21(4):519–30. doi: 10.1016/s1470-2045(19)30863-0 32171426

[20] van Ramshorst MS van der Voort A van Werkhoven ED Mandjes IA Kemper I Dezentjé VO. Neoadjuvant Chemotherapy With or Without Anthracyclines in the Presence of Dual Her2 Blockade for Her2-Positive Breast Cancer (Train-2): A Multicentre, Open-Label, Randomised, Phase 3 Trial. Lancet Oncol (2018) 19(12):1630–40. doi: 10.1016/s1470-2045(18)30570-9 30413379

[21] Meric-Bernstam F Hurwitz H Raghav KPS McWilliams RR Fakih M VanderWalde A. Pertuzumab Plus Trastuzumab for Her2-Amplified Metastatic Colorectal Cancer (Mypathway): An Updated Report From a Multicentre, Open-Label, Phase 2a, Multiple Basket Study. Lancet Oncol (2019) 20(4):518–30. doi: 10.1016/s1470-2045(18)30904-5 PMC6781620 30857956

[22] Gianni L Pienkowski T Im YH Tseng LM Liu MC Lluch A. 5-Year Analysis of Neoadjuvant Pertuzumab and Trastuzumab in Patients With Locally Advanced, Inflammatory, or Early-Stage Her2-Positive Breast Cancer (Neosphere): A Multicentre, Open-Label, Phase 2 Randomised Trial. Lancet Oncol (2016) 17(6):791–800. doi: 10.1016/s1470-2045(16)00163-7 27179402

[23] Wu Z Xu S Zhou L Yin W Lin Y Du Y. Clinical Significance of Quantitative Her2 Gene Amplification as Related to Its Predictive Value in Breast Cancer Patients in Neoadjuvant Setting. Onco Targets Ther (2018) 11:801–8. doi: 10.2147/OTT.S157634 PMC5818868 29497312

[24] Katayama A Miligy IM Shiino S Toss MS Eldib K Kurozumi S. Predictors of Pathological Complete Response to Neoadjuvant Treatment and Changes to Post-Neoadjuvant Her2 Status in Her2-Positive Invasive Breast Cancer. Modern Pathol (2021) 34(7):1271–81. doi: 10.1038/s41379-021-00738-5 PMC8216906 33526875

[25] Swain SM Ewer MS Viale G Delaloge S Ferrero JM Verrill M. Pertuzumab, Trastuzumab, and Standard Anthracycline- and Taxane-Based Chemotherapy for the Neoadjuvant Treatment of Patients With Her2-Positive Localized Breast Cancer (Berenice): A Phase Ii, Open-Label, Multicenter, Multinational Cardiac Safety Study. Ann Oncol: Off J Eur Soc Med Oncol (2018) 29(3):646–53. doi: 10.1093/annonc/mdx773 PMC5888999 29253081

[26] André F Ciruelos E Rubovszky G Campone M Loibl S Rugo HS. Alpelisib for Pik3ca-Mutated, Hormone Receptor-Positive Advanced Breast Cancer. N Engl J Med (2019) 380(20):1929–40. doi: 10.1056/NEJMoa1813904 31091374

[27] Hurvitz SA Martin M Symmans WF Jung KH Huang CS Thompson AM. Neoadjuvant Trastuzumab, Pertuzumab, and Chemotherapy Versus Trastuzumab Emtansine Plus Pertuzumab in Patients With Her2-Positive Breast Cancer (Kristine): A Randomised, Open-Label, Multicentre, Phase 3 Trial. Lancet Oncol (2018) 19(1):115–26. doi: 10.1016/s1470-2045(17)30716-7 29175149

[28] Fehrenbacher L Cecchini RS Geyer CE Jr Rastogi P Costantino JP Atkins JN. Nsabp B-47/Nrg Oncology Phase III Randomized Trial Comparing Adjuvant Chemotherapy With or Without Trastuzumab in High-Risk Invasive Breast Cancer Negative for Her2 by Fish and With Ihc 1+ or 2. J Clin Oncol: Off J Am Soc Clin Oncol (2020) 38(5):444–53. doi: 10.1200/jco.19.01455 PMC7007289 31821109

[29] Krystel-Whittemore M Xu J Brogi E Ventura K Patil S Ross DS. Pathologic Complete Response Rate According to Her2 Detection Methods in Her2-Positive Breast Cancer Treated With Neoadjuvant Systemic Therapy. Breast Cancer Res Treat (2019) 177(1):61–6. doi: 10.1007/s10549-019-05295-9 PMC6640097 31144151

[30] Meisel JL Zhao J Suo A Zhang C Wei Z Taylor C. Clinicopathologic Factors Associated With Response to Neoadjuvant Anti-Her2-Directed Chemotherapy in Her2-Positive Breast Cancer. Clin Breast Cancer (2020) 20(1):19–24. doi: 10.1016/j.clbc.2019.09.003 31806448

[31] Coudert BP Arnould L Moreau L Chollet P Weber B Vanlemmens L. Preoperative Systemic (Neo-Adjuvant) Therapy With Trastuzumab and Docetaxel for Her2-Overexpressing Stage Ii or Iii Breast Cancer: Results of a Multicenter Phase Ii Trial. Ann Oncol: Off J Eur Soc Med Oncol (2006) 17(3):409–14. doi: 10.1093/annonc/mdj096 16332965

[32] Coudert BP Largillier R Arnould L Chollet P Campone M Coeffic D. Multicenter Phase Ii Trial of Neoadjuvant Therapy With Trastuzumab, Docetaxel, and Carboplatin for Human Epidermal Growth Factor Receptor-2-Overexpressing Stage Ii or Iii Breast Cancer: Results of the Getn(a)-1 Trial. J Clin Oncol: Off J Am Soc Clin Oncol (2007) 25(19):2678–84. doi: 10.1200/jco.2006.09.9994 17515572

[33] Chumsri S Li Z Serie DJ Mashadi-Hossein A Colon-Otero G Song N. Incidence of Late Relapses in Patients With Her2-Positive Breast Cancer Receiving Adjuvant Trastuzumab: Combined Analysis of Ncctg N9831 (Alliance) and Nrg Oncology/Nsabp B-31. J Clin Oncol: Off J Am Soc Clin Oncol (2019) 37(35):3425–35. doi: 10.1200/jco.19.00443 PMC6900835 31622131

[34] Kim HK Park KH Kim Y Park SE Lee HS Lim SW. Discordance of the Pam50 Intrinsic Subtypes Compared With Immunohistochemistry-Based Surrogate in Breast Cancer Patients: Potential Implication of Genomic Alterations of Discordance. Cancer Res Treat (2019) 51(2):737–47. doi: 10.4143/crt.2018.342 PMC6473265 30189722

